# Parity-time symmetry in wavelength space within a single spatial resonator

**DOI:** 10.1038/s41467-020-16705-8

**Published:** 2020-06-25

**Authors:** Jiejun Zhang, Lingzhi Li, Guangying Wang, Xinhuan Feng, Bai-Ou Guan, Jianping Yao

**Affiliations:** 1 Guangdong Provincial Key Laboratory of Optical Fiber Sensing and Communications, Institute of Photonics Technology, Jinan University, Guangzhou, 511443 China; 2 Microwave Photonics Research Laboratory, School of Electrical Engineering and Computer Science, University of Ottawa, Ottawa, ON K1N 6N5 Canada

**Keywords:** Electrical and electronic engineering, Microwave photonics, Optoelectronic devices and components

## Abstract

We show a parity-time (PT) symmetric microwave photonic system in the optical wavelength space within a single spatial resonator, in which the gain and loss modes can perfectly overlay spatially but are distinguishable in the designated parameter space. To prove the concept, a PT-symmetric optoelectronic oscillator (OEO) in the optical wavelength space is implemented. The OEO has a single-loop architecture, with the microwave gain and loss modes carried by two optical wavelengths to form two mutually coupled wavelength-space resonators. The operation of PT symmetry in the OEO is verified by the generation of a 10-GHz microwave signal with a low phase noise of −129.3 dBc/Hz at 10-kHz offset frequency and small sidemodes of less than −66.22 dBc/Hz. Compared with a conventional spatial PT-symmetric system, a PT-symmetric system in the wavelength space features a much simpler configuration, better stability and greater resilience to environmental interferences.

## Abstract (web summary)

Most conventional PT-symmetric systems contain two physically separated modes. Here the authors proposed a PT-symmetric microwave optoelectronic oscillator, where the gain and loss modes are confined in a single spatial resonator, resulting in an increased structural simplicity, and long-term stability.

## Introduction

Parity-time (PT) symmetry has been an active topic and has attracted intensive research interests from the photonics community in the past few years^1–3^. As a special non-Hermitian quantum system, a PT-symmetric system has real eigenvalues, which counters the common intuition that real eigenvalues are only related to Hermitian observables^4^. The discovery of PT symmetry has led to a burst of studies in both microscopic and macroscopic systems, including but not limited to atomic^5^, electronic^6–8^, thermal^9^, photonic^10–19^ and optoelectronic systems^20,21^. Photonic techniques have been proven to be effective to implement PT symmetry, where the complex refractive index distribution of a photonic system can be used to emulate the PT-symmetric potential such that $U\left( r \right) = U^ \ast \left( { - r} \right)$, where * denotes complex conjugate and *r* is the position vector^1,3^. So far, experimental implementations of PT symmetry are bounded to one-dimensional space where *r* becomes a scalar, with most demonstrations focusing only on a PT-symmetric systems with two subspaces^6–11,14–21^ and a few being extended to multiple subspaces^12,13^. One-dimensional PT symmetry formed by two subspaces is the simplest form of PT symmetry as the energy flow between the subspaces is the key to achieve the complementary gain and loss modes. In addition, the parity transformation in a singular system, e.g., a single subspace, becomes meaningless.

Since the advent of implementing PT symmetry in photonics, a variety of optical devices and subsystems with novel functionalities emerge. For example, PT symmetry was employed for mode selection in a micro-ring laser by controlling the gain and loss interplay between two ring cavities^14,15^; PT symmetry was used to implement a coherent perfect absorber that can potentially realize extremely high modulation depth for future communications systems^16,17^; PT symmetry was used to implement optical nonreciprocity without the need of ferromagnetic materials and thus is suitable for large scale integration^18,19^. Recently, PT symmetric optoelectronic oscillators (OEOs) with two cross-coupled optoelectronic loops were demonstrated. By controlling the gain and loss interactions, a single-frequency microwave signal with an ultra-low phase noise was generated^20,21^. This was the first time that PT symmetry was introduced to microwave photonic systems to overcome the long existing mode-selection challenge, which has severely jeopardized the development and wide applications of OEOs^22–24^.

A significant common ground of implementing those PT-symmetric systems is that they consist of two spatially distributed subspaces, usually two independent resonators, to support the gain and loss supermodes. The successful realization of PT symmetry relies critically on the perfect matching of the geometry of the two resonators and the availability of techniques to control the gain, loss and mutual coupling between them. Compared to a Hermitian system, a redundant resonator in a PT-symmetric system makes it more precision demanding in terms of fabrication or construction and more susceptible to interferences especially for a macro system as the resonators may have vastly different localized perturbations. Hence, a simple question is if there is a technique to implement a PT-symmetric system with a single spatial resonator. Note that such a PT-symmetric system has only one subspace, energy flow between two subspaces in a classical PT-symmetric system seems intuitively impossible. Only very recently, optical PT-symmetric systems in a synthetic dimension created by the physical parameters of photons have been reported and experimentally demonstrated, where counterpropagating light waves in a single optical resonator were employed^25–29^. However, such implementations rely on complicated optical phenomena or techniques, such as on-chip stimulated Brillouin scattering effect^26,27^, Faraday effect^28^, and on-chip and in-resonator temporal modulation^29^, which make the PT-symmetric systems have low degree of freedom with very limited scalability. In addition, the technical significance of using nonspatial parameters of a photon to implement PT symmetry has not been explored.

Multiplexing is a well-established technique that is widely used in modern optical communications systems to increase the bandwidth, such as time-division multiplexing (TDM) and wavelength-division multiplexing (WDM). For TDM, an individual channel occupies a time slot and multiple channels are multiplexed in the optical traffic train. For WDM, an individual channel is modulated to one wavelength and multiple channels at multiple wavelengths are multiplexed and transmitted over an optical fiber^30,31^. TDM and WDM techniques are used to expand the capacity of a single optical fiber to that of multiple fibers, thus the spatial redundancy is reduced.

In this paper, we propose and experimentally demonstrated that PT symmetry can be implemented in a nonspatial parameter space, which is fundamentally different from any existing spatial PT-symmetric systems^14–21^ by transferring the spatial duplicity of the PT-symmetric subspaces into a nonspatial parameter space, and thus to reduce the system complexity by eliminating spatial duplicity and significantly improve the stability as compared with a spatially PT-symmetric system^14–21^. For experimental verification, we design a PT-symmetric OEO in a nonspatial parameter space of wavelength for high quality microwave signal generation. We demonstrate that PT symmetry is achievable between the two wavelength-space resonators (WSRs), leading to single-mode oscillation of the OEO. Our studies show that a wavelength-space PT-symmetric system, compared with a spatial-space PT-symmetric system with two spatially separated loops^20^, has higher stability, lower complexity, and smaller footprint. It also features great scalability due to the availability of other physical parameter space.

## Results

### Design concept

**Fig. 1:**
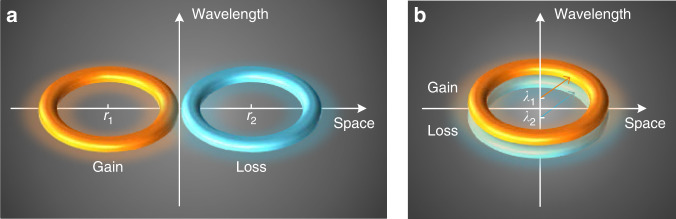
Comparison of a spatial PT-symmetric system and a PT-symmetric system in the parameter space of wavelength. **a** A conventional PT-symmetric system with binary spatial arrangement with two subspaces located at *r*_1_ and *r*_2_^20^; **b** the proposed PT-symmetric system in the parameter space of wavelength with two subspaces located at *λ*_1_ and *λ*_2_. The two subspaces are non-distinguishable in terms of spatial location, i.e., they fully overlap in space and the footprint is only half that of its spatial counterpart.

The design concept is illustrated in Fig. 1a, b in which a spatial and a nonspatial (such as one in the wavelength space) PT-symmetric systems are shown, respectively. As can be seen a conventional PT-symmetric system has a binary spatial arrangement with two separated subspaces while a nonspatial PT-symmetric system is in a parameter space of optical wavelength with two subspaces located at two optical wavelengths. In our demonstration, the two subspaces corresponding to two WSRs are implemented in a single OEO loop with microwave modes modulated on two optical wavelengths. Since two optical wavelengths are employed, the system has two optoelectronic resonators in the parameter space of optical wavelength (see Supplementary Note 1). To achieve PT symmetry, the WSRs are designed to be mutually coupled with independently controllable mode spacing and round-trip gains, but physically overlap in spatial space.

### System design

**Fig. 2:**
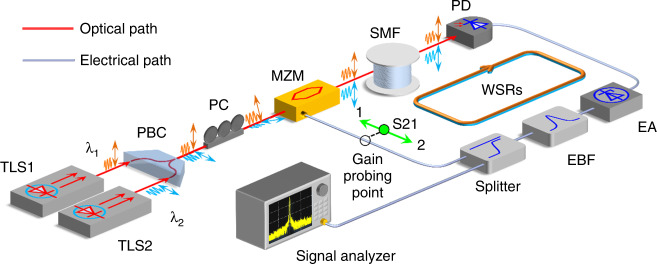
Experimental implementation of a PT-symmetric OEO in the wavelength space. The system consists of a single optical and electrical hybrid spatial loop, in which two optical carriers of different wavelengths are used to allow the propagation of the microwave eigenmodes through the delay fiber. TLS tunable laser source; PBC polarizer beam combiner; PC polarization controller; MZM Mach-Zehnder modulator; SMF single-mode fiber; PD photodetector; EA electrical amplifier; EBF electrical bandpass filter; WSR wavelength-space resonator.

The schematic of the wavelength-space PT-symmetric system is shown in Fig. 2. It consists of two tunable laser sources (TLSs), a Mach-Zehnder modulator (MZM), a photodetector (PD), an electrical amplifier (EA), a long single-mode fiber (SMF), an electrical bandpass filter (EBF), and a microwave power splitter. The two TLSs are used to generate two optical carriers with different wavelengths that are combined at a polarizer beam combiner (PBC) and sent to the MZM via a polarization controller (PC). The polarization directions of the optical carriers are orthogonal and are aligned with the two principal axes of the PBC to maximize the combination efficiency. Note that the MZM has a built-in polarizer, the tuning of the PC will change the polarization direction alignment between the polarization directions of the carriers and the principal axis of the MZM, thus changes the powers of the optical carriers injected into the OEO loop. In this way, the optical power ratio between the two optical carriers recirculating in the loop can be continuously tuned, while the total optical power applied to the MZM remains constant (see Supplementary Note 2). The two optical carriers are modulated by microwave modes at the MZM, which then travel through a 10-km SMF and are then detected at the PD and converted back to the electrical domain. After electrical amplification by the EA, the electrical signal is split into two parts by a 3-dB power splitter, with 50% of the output fed back to the MZM after the EBF to close the OEO loop and the other 50% sent to an electrical spectrum analyzer (ESA) for signal quality analysis. The combined operation of the PD, the EA, the EBF, the power splitter, and the MZM corresponds to a microwave 2 × 2 coupler that allows the coupling between the microwave eigenmodes modulated on the two optical carriers (see Supplementary Note 3) with tunable coupling ratio.

### Theory

Due to the chromatic dispersion of the SMF, the eigenfrequency of the local mode in a WSR is dependent on the optical carrier wavelength, which is given by:

1 $$\omega _m = \frac{{2m\pi }}{{\tau _0 + D\left( {\lambda - \lambda _0} \right)}},$$

where *ω*_*m*_ is the angular frequency of the *m*-th order eigenmode, *τ*_0_ is the round-trip time delay of a reference carrier wavelength of *λ*_0_, *D* is the dispersion coefficient of the SMF, and *λ* is the optical carrier wavelength from the TLSs. Since the two optical carriers are of different wavelengths, the PT symmetry across all modes of the two WSRs are not achievable. However, with the adoption of the narrowband EBF, PT symmetry can be achieved among the eigenmodes within the passband if the passband width of the electrical filter $\Delta f \ll 1/\left( {\Delta \lambda \cdot D} \right)$, and if the wavelength difference between the two carriers is (see Supplementary Note 1):

2 $$\Delta \lambda = \frac{n}{{f_{\mathrm{c}} \cdot D}},$$

where *n* is an integer and *f*_*c*_ is the central passband frequency.

With PT symmetry, the coupled differential equations of the wavelength-space subsystems can be written as:

3 $$i\frac{d}{{dt}}\left( \begin{array}{l}a\\ b\end{array} \right) = \left( {\begin{array}{*{20}{c}} {\omega ^{\left( 1 \right)} + i\gamma ^{\left( 1 \right)}} & { - \kappa } \\ { - \kappa } & {\omega ^{\left( 2 \right)} + i\gamma ^{\left( 2 \right)}} \end{array}} \right)\left( \begin{array}{l}a\\ b\end{array} \right),$$

where *a* and *b* are the amplitudes of the localized eigenmodes in the two WSRs, of which the eigenvalues are *ω*^(1)^ and *ω*^(2)^; *γ*^(1)^ and *γ*^(2)^ are the gain or loss coefficients of the two WSRs and *κ* is the coupling coefficient between them. A detailed calculation of the eigenvalues, the gain, the loss, and the coupling coefficients can be found in the Supplementary Notes 1–3. With PT symmetry, we have *ω*^(1)^ = *ω*^(2)^ = *ω*_*m*_ and *γ*^(1)^ = −*γ*^(2)^ = *γ*_*m*_. The eigenfrequencies of the supermodes in the PT-symmetric system can be calculated to be (See Supplementary Note 4)

4 $$\omega _m^{\left( {1,2} \right)} = \omega _m \pm \sqrt {\kappa ^2 - \gamma _m^2}.$$

PT symmetry enhances the gain contrast between the modes with the highest and the second highest loop gain within the EBF bandwidth by a factor of $F = \sqrt {\gamma _m^2 - \gamma _n^2} /\left( {\gamma _m - \gamma _n} \right)$. It thus enables single-mode oscillation of the WSR-based PT-symmetric OEO, which would otherwise be impossible since there are approximately 1000 modes within the passband of the EBF.

Furthermore, a PT-symmetric OEO in the wavelength space also implies higher operation stability, as compared with a spatial PT-symmetric OEO under environmental disturbances such as temperature change or mechanical vibrations. Perturbations imposed on a PT-symmetric system can be decomposed into common mode and differential mode signals with respect to the two subsystems. For WSR-based PT-symmetric OEO, the temperature or vibration disturbance affects two subsystems indiscriminately, the perturbed Hamiltonian of the system is thus given by:

5 $$H = \left( {\begin{array}{*{20}{c}} {\omega _m + i\gamma _m + \varepsilon _{\omega _m^{\left( 1 \right)}}} & { - \kappa } \\ { - \kappa } & {\omega _m - i\gamma _m + \varepsilon _{\omega _m^{\left( 1 \right)}}} \end{array}} \right),$$

where $\varepsilon _{\omega _m^{\left( 1 \right)}}$ is the localized eigenfrequency perturbation induced by temperature change or vibrational interferences^32^ (see Supplementary Notes 5–7). The corresponding perturbed eigenfrequency of the supermodes is $\omega _m = - \varepsilon _{\omega _m^{\left( 1 \right)}}$, indicating that in the presence of environmental interferences, no eigenfrequency bifurcation will appear to affect the stability of PT symmetry. Instead, the eigenfrequency of both the gain and loss supermodes will drift at the same rate, ensuring that PT symmetry is always preserved. Though temperature can also affect the dispersion coefficient of the SMF and thus affect the PT symmetry, it is proven that such effect is small and negligible within a practical application scenario.

### Open-loop analysis

**Fig. 3:**
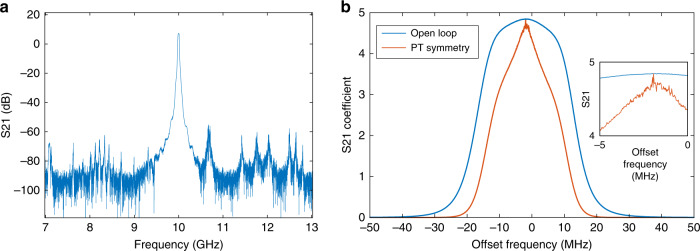
The open-loop gain spectrum. **a** Gain spectrum measurement by a VNA connected at the gain probing point; **b** Zoom-in view of the gain spectrum at 10 GHz (blue) and the calculated gain profile (red) after gain contrast enhancement with PT symmetry. Inset: zoom-in view of the enhanced ripples.

We first measure the open-loop frequency response of the OEO loop at the gain probing point (shown in Fig. 2) by opening the feedback loop and adding a vector network analyzer (VNA) between the MZM and the power splitter. The EA is employed to provide a gain to make the open-loop response greater than unity at certain frequency band, so that the modes within the frequency band can exceed the oscillation threshold. Figure 3a shows the measured open-loop frequency response of the OEO loop, in which the EBF with a central frequency of 10 GHz and a bandwidth of 28 MHz is employed. The frequency response (blue) has a smooth profile, as shown in Fig. 3b. The calculated gain profile (red) with gain contrast enhancement due to PT symmetry is also shown in Fig. 3b. Without PT symmetry, it is estimated that there are about 1000 longitudinal modes within the passband. Thus, single-mode oscillation would not be possible without the adoption of PT symmetry in the system.

### Performance evaluation

**Fig. 4:**
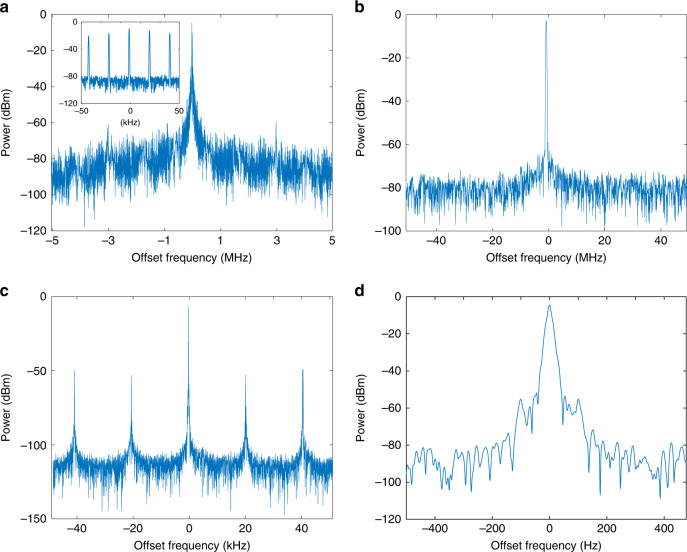
The electrical spectra of the microwave signals generated by the wavelength-space PT-symmetric OEO. The spectra are measured at a central frequency at 10 GHz. **a** Multimode oscillation spectrum measured by an ESA with a resolution bandwidth (RBW) of 3 MHz. Inset: zoom-in view of the multimode spectrum showing multiple modes with comparable amplitudes; single-mode oscillation spectra measured with RBWs of **b** 3 MHz, **c** 100 kHz and **d** 9 Hz. The spectrum in (**c**) shows a dominating mode with a sidemode suppression ration of 46.75 dB.

Then, we close the OEO loop and microwave oscillation will start. We analyze the generated microwave signal using an ESA. If only one TLS is turned on, the system is a single-loop OEO without PT symmetry. Due to the wide bandwidth of the EBF, multimode oscillation is resulted. The spectrum of the generated microwave signal is shown in Fig. 4a and the inset shows the multiple modes. To achieve single-mode oscillation, PT symmetry must be enabled, which is done by switching on the two TLSs. The wavelength spacing is calculated based on Eq. (2). The light waves from the TLSs are orthogonally polarized and are combined at the PBC. Thanks to the built-in polarizer in the MZM, the tuning of the PC can result in a change in the power ratio between the two wavelengths in the feedback loop, i.e., by tuning the PC, the WSR corresponding to one wavelength can have a round-trip gain, while the WSR corresponding to the other wavelength can have a round-trip loss. In this way, PT symmetry is achieved between the two WSRs (see Supplementary Movie). The small gain ripples observed from the open-loop gain spectrum are enhanced to large ripples, as illustrated in Fig. 3b. The spectrum of the generated signal with a 100-MHz measurement span in Fig. 4b shows that a single longitudinal-mode oscillation is achieved near the frequency of 10 GHz. Figure 4c, d shows the spectrum with two different measurement spans of 100 kHz and 1 kHz, respectively. The longitudinal-mode spacing of the OEO is about 20 kHz and the oscillating mode is 46.75 dB greater than the highest sidemode. Thanks to the single spatial loop architecture, we observe that the OEO operates stably without mode hopping within 1 h of monitoring time without any isolation from the environmental interferences in a laboratory environment. Our analysis shows that the PT-symmetric system in the wavelength space can be over 1000 times more resilient to environmental perturbation as compared with its spatial counterpart (see Supplementary Notes 5–7).

**Fig. 5:**
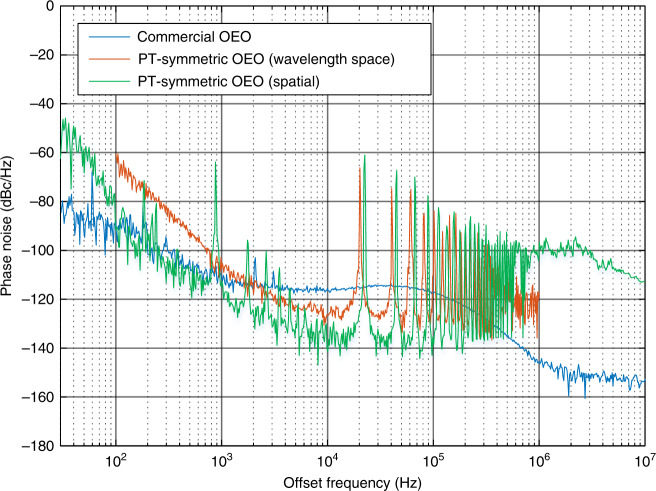
Measured phase noise of the generated microwave signal (red). For comparison, the phase noises of microwave signals generated by a commercial microwave source (blue) and a spatially separated dual-loop OEO with a 9.1-km loop length (green) are also shown.

Figure 5 shows the measured phase noise of the generated microwave signal. The phase noise is −129.3 dBc/Hz at an offset frequency of 10 kHz with sidemodes lower than −66.22 dBc/Hz, which verifies the effectiveness of the mode-selection mechanism in the WSR-based PT-symmetric OEO. For comparison, the phase noises of two signals generated by a commercial microwave signal generator (Agilent E8254A) and a spatially separated dual-loop PT-symmetric OEO^20^ are also shown in Fig. 5. At an offset frequency of 10 kHz, the phase noise of the WSR-based PT-symmetric OEO is 13.4 dB lower than that of the commercial microwave source, but 13.0 dB higher than that of a spatially separated dual-loop OEO due to the higher noise floor of the measurement instrument that we use in this experiment. Since the phase noise performance of an OEO is mainly determined by the length of the SMF within the loop, we estimate that the actual phase noise of the OEO reported here is around −140 dBc/Hz at 10 kHz offset frequency if a measurement instrument with a sufficiently low noise floor is used^22,23^.

## Discussion

In conclusion, we have proposed and demonstrated that PT symmetry can be implemented in the parameter space of optical wavelength, to eliminate the requirement for physically separated spatial subsystems in a conventional PT-symmetric system. An OEO with two optical carriers was implemented to validate the concept. Single-mode oscillation was realized with the single-spatial-loop OEO, in which a low phase noise microwave signal was generated with a high sidemode suppression ratio. The WSR-based PT-symmetric OEO is over 1000 times more resilient to environment interference, and it is much easier to implement due to the structural simplicity and compactness compared to a conventional spatial-space PT-symmetric system^20^. By implementing coupled subspaces in the wavelength space, the PT-symmetric OEO features a single-closed loop, which counters common knowledge that a PT-symmetric system must be an open system that interacts with the environment^1^.

The concept of constructing PT-symmetric systems in physical parameter spaces other than those only based on spatial arrangements has enabled the possibility toward a significantly enhanced performance and functionality. For example, in an integrated PT-symmetric system, spatial compactness due to the implementation in a nonspatial parameter space can increase the operation stability, double the integration density and reduce the cost; the high degree of freedom in wavelength space and the availability of wavelength switching and tuning techniques developed for optical communications systems guarantee the high performance and high scalability of a PT-symmetric system; the multidimensional parameter space of location, polarization, transverse mode, angular momentum, and wavelength can be used to implement a complex PT-symmetric network, which enables the controllable multidimensional energy flow and may find great applications in future neuro-networks.

## Methods

### Implementation of the OEO

The OEO is implemented using commercial off-the-shelf optical and optoelectronic components. The light waves from TLS1 and TLS2 are generated by a four-port laser source (Keysight N7714A). The MZM (MX-LN-40) has a bandwidth of 30 GHz from Photline Technologies. The PD (XPDV2120RA) has a bandwidth of 50 GHz from U2t. The EA is implemented by cascading two EAs (SHF 806E) to provide a total electrical gain of over 50 dB. The EBF (GBPF100-28M-SMF) has a bandwidth of 28 MHz at a center frequency of 10 GHz from GWAVE.

### Performance evaluation of the OEO

The spectrum and the phase noise of the microwave signal generated by the OEO are both analyzed by a signal analyzer (Keysight N9040B). The open-loop frequency response of the OEO loop is measured using a VNA (Keysight N5224A).

## Supplementary information

SUPPLEMENTARY INFO

## Data Availability

All the data supporting the findings of this study are available from the corresponding authors upon request.

## References

[1] El-Ganainy R (2018). Non-Hermitian physics and PT symmetry. Nat. Phys..

[2] Özdemir Ş, Rotter S, Nori F, Yang L (2019). Parity–time symmetry and exceptional points in photonics. Nat. Mater..

[3] Feng L, El-Ganainy R, Ge L (2017). Non-Hermitian photonics based on parity–time symmetry. Nat. Photonics.

[4] Bender CM, Boettcher S (1998). Real spectra in non-Hermitian Hamiltonians having P T symmetry. Phys. Rev. Lett..

[5] Hang C, Huang G, Konotop VV (2013). P T symmetry with a system of three-level atoms. Phys. Rev. Lett..

[6] Schindler J, Li A, Zheng MC, Ellis FM, Kottos T (2011). Experimental study of active LRC circuits with PT symmetries. Phys. Rev. A.

[7] Assawaworrarit S, Yu X, Fan S (2017). Robust wireless power transfer using a nonlinear parity–time-symmetric circuit. Nature.

[8] Chen P-Y (2018). Generalized parity–time symmetry condition for enhanced sensor telemetry. Nat. Electron..

[9] Li Y (2019). Anti–parity-time symmetry in diffusive systems. Science.

[10] Regensburger A (2012). Parity–time synthetic photonic lattices. Nature.

[11] Peng B (2014). Parity–time-symmetric whispering-gallery microcavities. Nat. Phys..

[12] Hodaei H (2017). Enhanced sensitivity at higher-order exceptional points. Nature.

[13] Perez-Leija A (2013). Coherent quantum transport in photonic lattices. Phys. Rev. A.

[14] Hodaei H, Miri M-A, Heinrich M, Christodoulides DN, Khajavikhan M (2014). Parity-time–symmetric microring lasers. Science.

[15] Feng L, Wong ZJ, Ma R-M, Wang Y, Zhang X (2014). Single-mode laser by parity-time symmetry breaking. Science.

[16] Wong ZJ (2016). Lasing and anti-lasing in a single cavity. Nat. Photonics.

[17] Wan W (2011). Time-reversed lasing and interferometric control of absorption. Science.

[18] Zhu X, Feng L, Zhang P, Yin X, Zhang X (2013). One-way invisible cloak using parity-time symmetric transformation optics. Opt. Lett..

[19] Sounas DL, Fleury R, Alù A (2015). Unidirectional cloaking based on metasurfaces with balanced loss and gain. Phys. Rev. Appl..

[20] Zhang J, Yao JP (2018). Parity-time–symmetric optoelectronic oscillator. Sci. Adv..

[21] Liu Y (2018). Observation of parity-time symmetry in microwave photonics. Light. Sci. Appl..

[22] Yao XS, Maleki L (1996). Optoelectronic microwave oscillator. JOSA B.

[23] Yao XS, Maleki L (1996). Optoelectronic oscillator for photonic systems. IEEE J. Quantum Electron..

[24] Li W, Yao J (2012). A wideband frequency tunable optoelectronic oscillator incorporating a tunable microwave photonic filter based on phase-modulation to intensity-modulation conversion using a phase-shifted fiber Bragg grating. IEEE Trans. Microw. Theory Tech..

[25] Yuan L, Lin Q, Xiao M, Fan S (2018). Synthetic dimension in photonics. Optica.

[26] Lai Y-H, Lu Y-K, Suh M-G, Yuan Z, Vahala K (2019). Observation of the exceptional-point-enhanced Sagnac effect. Nature.

[27] Zhang F, Feng Y, Chen X, Ge L, Wan W (2020). Synthetic Anti-PT Symmetry in a Single Microcavity. Phys. Rev. Lett..

[28] Hokmabadi MP, Schumer A, Christodoulides DN, Khajavikhan M (2019). Non-Hermitian ring laser gyroscopes with enhanced Sagnac sensitivity. Nature.

[29] Dutt A (2020). A single photonic cavity with two independent physical synthetic dimensions. Science.

[30] Kikuchi K (2016). Fundamentals of coherent optical fiber communications. J. Lightwave Technol..

[31] Agrawal, G. P. *Fiber-optic communication systems*. Vol. 222 (John Wiley & Sons, 2012).

[32] Kersey AD (1997). Fiber grating sensors. J. lightwave Technol..

