# Supplementary material for: Parity-time symmetry in wavelength space within a single spatial resonator
Source: Nat Commun. 2020 Jun 25;11:3217. doi: 10.1038/s41467-020-16705-8 (PMC7316734; doi:10.1038/s41467-020-16705-8)
Supplement: Supplementary file 1 — SUPPLEMENTARY INFO [file 41467_2020_16705_MOESM1_ESM.pdf]

Supplementary Information for

Parity-time symmetry in wavelength space within a single spatial resonator

Zhang et al.

Supplementary Note 1. WAVELENGTH-SPACE RESONATORS AND EIGENMODES

In an optical and electrical hybrid loop or resonator, the round-trip time is a summation of the time delays introduced by the electrical and optical paths. The time delay of the electrical path is much smaller than that of the optical path, and is independent of the optical carrier wavelength, hence it is ignored in our analysis. The time delay of the optical path is mainly due to the long optical fiber which is usually a single mode fiber (SMF). The total round-trip time delay can be approximately expressed as

$$\tau(\lambda) = \tau_0 + D \cdot (\lambda - \lambda_0) \quad (1)$$

where λ is the carrier wavelength; τ_0 is the time delay of a reference wavelength λ_0 as it travels through the SMF and D is the dispersion coefficient of the SMF. Note that the hybrid resonator does not contain a closed loop for the optical carrier. At the photodetector, the microwave signal modulated on the optical carrier will be detected and is directed to the MZM via the RF input port to form a closed microwave loop. Hence, the optical carrier only plays a role to carry the microwave signal to enable its transmission in the optical fiber and the value of wavelength can be controlled to adjust the round-trip time, or the eigenfrequency of the resonator in the microwave regime. The free spectral range (FSR) of the resonator is given by

$$FSR(\lambda) = \frac{1}{\tau(\lambda)} \quad (2)$$

If two wavelengths are employed, two sets of eigenfrequencies will be achieved due to chromatic dispersion of the fiber, which corresponds to two sets of localized eigenfrequencies of two resonators in the wavelength space. Perfect match of all eigenfrequencies between the two wavelength space resonators (WSRs, denoted as WSR1 and WSR2 for the equivalent gain and loss resonators, respectively) cannot be achieved due to the different FSRs. We use a microwave bandpass filter in the electrical path, which has as a central frequency of f_c and a bandwidth of f_{BW} to ensure the symmetry of real part of the eigenfrequencies within the passband. As illustrated in Supplementary Figure 1, the FSR mismatch within the passband should be small and negligible if

$$\left|\frac{f_{\mathrm{BW}}}{FSR(\lambda_1)}-\frac{f_{\mathrm{BW}}}{FSR(\lambda_2)}\right| \ll 1 \tag{3}$$

or

$$D\cdot f_{\mathrm{BW}}\cdot\Delta\lambda \ll 1 \tag{4}$$

where $\Delta\lambda = |\lambda_1-\lambda_2|$ is the wavelength difference between the two optical carriers and f_{BW} is the bandwidth of the electrical filter. Such a condition can be easily satisfied by using two wavelengths with a small wavelength separation and a narrowband filter, as shown in Supplementary Figure 1.

Although Eq. (4) shows that small wavelength spacing between the optical carriers is more preferable for the WSRs to have a negligible FSR difference, the exact carrier-wavelength spacing should be properly chosen according to the dispersion coefficient of the optical fiber, so that the eigenfrequencies of one of the WSRs can be aligned to those of the other within the microwave filter passband. To quantify carrier-wavelength spacing, we consider that the system oscillates at the central frequency of the electrical bandpass filter f_{c}, which is also the frequency of the two degenerate eigenmodes $\omega_m^{(1)}$ and $\omega_n^{(2)}$, i.e., the *m*-th and *n*-th order modes of WSR1 and WSR2, respectively. The two eigenmodes can be expressed as

$$\begin{aligned}\omega_m^{(1)} &= \frac{2m\pi}{\tau(\lambda_1)},\\ \omega_n^{(2)} &= \frac{2n\pi}{\tau(\lambda_2)}\end{aligned} \tag{5}$$

By letting $\omega_m^{(1)} = \omega_n^{(2)}$, we get

$$\Delta\lambda = \frac{m-n}{D\cdot f_c} \tag{6}$$

Since $(m-n)$ is an integer, valid wavelength spacings are a set of discrete values that are inversely proportional to the product of the oscillation frequency and fiber dispersion coefficient.

In our experiment, the two WSRs are used to form an oscillator that support single-mode oscillation at a frequency of 10 GHz. The dispersion coefficient of the long fiber (10-km SMF) is 170 ps/nm. The wavelength spacing is 2.353 nm, where we choose $(m-n)=4$. Such a wavelength spacing can be implemented by using high accuracy tunable laser sources (TLSs). Moreover, the bandwidth of the electrical filter is $f_{\mathrm{BW}} = 20\ \mathrm{MHz}$, substitute this value to Eq. (4), we have $D\cdot f_{\mathrm{BW}}\cdot\Delta\lambda = 0.008 \ll 1$. Such an alignment accuracy is higher than those reported in (*1*-

3), which indicates that parity-time (PT) symmetry can be achieved across all modes within the electrical filter passband.

Supplementary Note 2. TUNING OF ROUND-TRIP GAIN IN A WSR

In addition to the alignment of eigenfrequency, which is related to the real part of the potential function in a PT-symmetric system, the gain and loss tuning the WSR is also critical as it is related to the symmetry of the imaginary part of the potential. A photonic or microwave PT-symmetric system requires that the gain and loss coefficients are of the same magnitude in the two subsystems, i.e., the gain coefficient of one of the WSRs is equal to the loss coefficient of the other. In our system, the gain and loss coefficient adjustment is achieved by changing the powers of the two optical carriers.

In a microwave path, the gain or loss coefficient is also known as the S21 parameter, which is measured by the ratio between the microwave power returned from the path and that injected into the path. Here, we choose a probing point for the round-trip gain between the MZM and the microwave splitter, which is experimentally measured by a microwave network analyzer, as shown in Supplementary Figure 2.

First, we assume the MZM is biased at the quadrature point and an electrical signal given by $e(t)=V_{\text{in}}\cos(\omega t)$ is applied to the MZM. with an amplitude of V_{in}. The electrical field of the electrical signal can be given as $e(t)=V_{\text{in}}\cos(\omega t)$; where V_{in} and ω are the amplitude and angular frequency of the electrical signal, respectively. Then, the optical power at the output of the MZM is given by

$$P_{\text{out}}^{(\text{o})}=\frac{1}{2}\alpha_{\text{MZM}}P_{\text{in}}^{(\text{o})}\left[1-\sin\left(\frac{\pi}{V_\pi}e(t)\right)\right] \tag{7}$$

where $P_{\text{in}}^{(\text{o})}$ is the power of the optical carrier that is launched into the MZM; α_{MZM} and V_π are the insertion loss and the half-wave voltage of the MZM, respectively. The modulated signal then travels through the SMF and is detected at the photodetector (PD). The photocurrent at the output of the PD is given by

$$\begin{aligned} i_{\text{PD}}(t) &= \alpha_{\text{SMF}}\Re P_{\text{out}}^{(\text{o})} \\ &= \frac{1}{2}\alpha_{\text{o}}\Re P_{\text{in}}^{(\text{o})}\left[1-\sin\left(\frac{\pi}{V_\pi}e(t)\right)\right] \end{aligned} \tag{8}$$

where $\Re$ is the responsivity of the PD; α_{SMF} is the insertion loss of the SMF and $\alpha_{\text{o}}=\alpha_{\text{MZM}}\alpha_{\text{SMF}}$ is the total optical loss. The photocurrent from the PD is then converted to a voltage signal by a transimpedance electrical amplifier (EA),

$$V_{\mathrm{PD}}\left(t\right)=R_{\mathrm{EA}}i_{\mathrm{PD}}\left(t\right)$$
$$=-\alpha_{\mathrm{o}}R_{\mathrm{EA}}\Re P_{\mathrm{in}}^{(\mathrm{o})}J_1\left(\frac{\pi V_{\mathrm{in}}}{V_\pi}\right)\cos\left(\omega t\right) \tag{9}$$

where R_{EA} is the load resistance of the EA and J_1 is the first order Bessel function of the first kind. It should be noted that in Eq. (9), we apply Jacobi-Anger expansion to the amplitude-modulated signal and ignore all DC and higher order harmonic components, which is the case in our proposed system due to the existence of a DC block in the EA and the incorporation of the EBF to filter out undesirable frequencies. The signal becomes a single-tone signal with a frequency equal to that of the input signal at the probing point. We can calculate the electrical power, given by

$$P_{\mathrm{PD}}^{(\mathrm{e})}=\frac{\tilde{V}_{\mathrm{PD}}^2}{2R_{\mathrm{EA}}}=\frac{1}{2}\alpha_{\mathrm{o}}^2R_{\mathrm{EA}}\Re^2P_{\mathrm{in}}^{(\mathrm{o})^2}J_1^2\left(\frac{\pi V_{\mathrm{in}}}{V_\pi}\right) \tag{10}$$

where $\tilde{V}_{\mathrm{PD}}$ is the amplitude of the electrical signal that is sent to the EA. The signal is then amplified by the EA, experiences losses from the splitter and the EBF and finally reaches the probing point. The gain or loss coefficient is given by

$$G_{\mathrm{loop}}=\frac{\alpha_{\mathrm{e}}G_{\mathrm{A}}P_{\mathrm{PD}}^{(\mathrm{e})}}{V_{\mathrm{in}}^2/\left(2R_{\mathrm{MZM}}\right)}=\alpha_{\mathrm{e}}G_{\mathrm{A}}\alpha_{\mathrm{o}}^2R_{\mathrm{EA}}^2\Re^2P_{\mathrm{in}}^{(\mathrm{o})^2}\cdot J_1^2\left(\pi V_{\mathrm{in}}/V_\pi\right)/V_{\mathrm{in}}^2 \tag{11}$$

where α_{e} is the total electrical insertion loss contributed by the splitter and the EBF and G_{A} is the gain factor of the EA. We assume that the load resistance of the MZM is the same as that of the EA. It can be seen from Eq. (11) that the round trip gain is proportional to the gain and loss factors contributed by the electrical path, and to the square of those contributed by the optical path. The term $J_1^2\left(\pi V_{\mathrm{in}}/V_\pi\right)/V_{\mathrm{in}}^2$ represents a saturation effect induced by the nonlinearity of the electro-optic intensity modulation (*4*) that is only prominent when the amplitude of the signal is comparable to the half-wave voltage V_π. To allow the gain and loss to be tunable, we observe that the gain or loss coefficient of the loop is related to the square of the power of the optical carrier. When two optical carriers are used, the loop gain of each subsystem can be tuned by changing the optical power of that individual carrier.

The essence of implementing PT symmetry is to adjust the gain difference between two WSRs such that when the system reaches equilibrium, one loop will have a net gain and the other will have a net loss. According to the discussion above, this can be implemented by changing the optical power ratio between the two carriers. We use a polarization beam combination scheme to adjust such power ratio, while the total powers of the two carriers are maintained nearly constant. This will guarantee that the PD works at its optimum input power level (*4*).

The principle of the polarization beam combination scheme is illustrated in Supplementary Figure 3. The two linearly polarized optical carriers of different wavelengths are generated by two tunable laser sources (TLSs) with equal power of $P_0^{(o)}$ and orthogonal polarization, which are combined at a polarization beam combiner (PBC). The polarization controller (PC) following the PBC is configured such that it does not vary the linear polarizations of the optical carriers, but only rotates the polarization directions by an angle of θ. This can be achieved by ensuring that the optical axes of the equivalent quarter wave plates are aligned to the polarization directions of the optical carriers, and that the optical axes of the equivalent half wave plate intersect with the polarization directions of the optical carriers with an angle of $\theta/2$. We assume that the direction of the polarizer integrated in the MZM assembly is aligned with carrier λ_1 before rotation. The optical power of the two carriers travelling through the PC, the polarizer and finally reaching the MZM assembly is given by

$$P_{\lambda_1} = P_0^{(o)} \cos^2(\theta) \tag{12}$$

$$P_{\lambda_2} = P_0^{(o)} \sin^2(\theta) \tag{13}$$

substituting to (11), the gain and loss coefficients of the two WSRs G_{λ_1} and G_{λ_2} become

$$G_{\lambda_1} = G_{\max} \cdot \cos^2(\theta) \tag{14}$$

$$G_{\lambda_2} = G_{\max} \cdot \sin^2(\theta) \tag{15}$$

$$G_{\max} = \alpha_e G_A \alpha_o^2 R_{EA}^2 \Re^2 P_0^{(o)^2} \cdot J_1^2\left(\pi V_{in}/V_\pi\right)/V_{in}^2 \tag{16}$$

where $G_{\max}$ is the maximum round-trip gain of a WSR, which is achieved when the carrier polarization is perfectly aligned to that of the polarizer. For PT symmetry, it is required that the two WSRs have gain and loss coefficients of the same magnitude, i.e., $G_{\lambda_1} \cdot G_{\lambda_2} = 1$, we solve that

$$\theta = \pm \arcsin\left(\frac{2}{G_{\max}}\right) \tag{17}$$

To ensure that a solution exists for θ, $G_{\max}$ must be greater than 2. The two solutions indicate that the polarization rotation angle of θ can be performed on either optical carrier, and the other will be rotated by $\theta + 180^\circ$, which leads to the identical PT-symmetry condition.

Supplementary Note 3. COUPLING BETWEEN WSRS

In addition to the match of eigenfrequencies, the gain and loss coefficients, the coupling between the WSRs are also mandatory for a PT-symmetric system (*2*).

In the proposed wavelength-space PT-symmetric optoelectronic oscillator (OEO), electrical signals in the two WSRs are launched into the PD simultaneously. Signal amplitude addition of the two signals in the two WSRs is automatically achieved at the output of the PD. The combined signal then propagates through the electrical path that is shared by the two WSRs and is applied to the MZM. It can be seen from Eq. (7) that both optical carriers are modulated with the same modulation depth $P_{\text{in}}^{(\text{o})} \big/ P_{\text{out}}^{(\text{o})}$, i.e., the signal is distributed into the two WSRs. From a microwave perspective, signals are combined and distributed indistinctively from and to the WSRs. Thus, the operation of the electrical path is equivalent to a microwave coupler with a coupling ratio of 1:1, as illustrated in **Supplementary Note** 4. Compared with a regular passive coupler, the incorporation of the EA in the electrical path can result in a negative insertion loss due to signal amplification. In the equivalent coupler, the powers of the optical carriers do not affect the coupling ratio, but the round-trip gain of the respective WSR.

Supplementary Note 4. PT SYMMETRY BASED ON TWO WSRS

A PT-symmetric system consists of two coupled cavities. The coupled differential equations of the system are given by

$$i\frac{d}{dt}\begin{pmatrix} a \\ b \end{pmatrix} = \begin{pmatrix} \omega^{(1)} + i\gamma^{(1)} & -\kappa \\ -\kappa & \omega^{(2)} + i\gamma^{(2)} \end{pmatrix}\begin{pmatrix} a \\ b \end{pmatrix} \tag{18}$$

where $\omega^{(1)}$ and $\omega^{(2)}$ are the eigenfrequencies of the two WSRs without gain, loss or coupling; a and b are the amplitude of the eigenmodes in the PT-symmetric system; $\gamma^{(1)}$ and $\gamma^{(2)}$ are the gain or loss coefficients of the two WSRs and κ is the coupling between the two WSRs. In a PT-symmetric system, it is required that $\omega^{(1)} = \omega^{(2)} = \omega_m$ and $\gamma^{(1)} = -\gamma^{(2)} = \gamma_m$, where m denotes the order of the primary eigenmode that is within the passband of the EBF. The eigenfrequencies of the PT-symmetric system can be given by

$$\omega_m^{(1,2)} = \omega_m \pm \sqrt{\kappa^2 - \gamma_m^2} \tag{19}$$

Considering a primary mode (*m*-th order) and a secondary mode (*n*-th order) with net loop gain coefficients of γ_m and γ_n, which are respectively the modes with the highest and the second highest net loop gain among all modes, the gain different between the *m*-th and *n*-th order modes with PT symmetry can be calculated by (*2*)

$$\Delta g_{\text{PT}} = \sqrt{\gamma_m^2 - \gamma_n^2} \tag{20}$$

Note that a single WSR system without PT symmetry is given by $\Delta g = \gamma_m - \gamma_n$. The WSR-based PT-symmetric system provides an enhanced gain difference between the primary and the secondary modes, thus single-mode oscillation can be easily achieved simply by using the

enhanced ripples on the frequency response of the WSRs. The single-mode oscillation can be used to verify the operation of PT symmetry in the wavelength space.

Supplementary Note 5. CARRIER WAVELENGTH PERTURBATION

In the proposed architecture, the two WSRs are formed by two optical carriers generated by two TLSs. As discussed in the previous Section, the two sets of eigenmodes corresponding to the two WSRs are the essence of the coupled subsystems required in a PT-symmetric system. In WSRs, the eigenmodes are tuned by tuning the wavelengths of the optical carriers, in contrast to those of the spatial PT-symmetric systems where the eigenmodes are tuned by the lengths of the spatial loops. Hence, these two kinds of PT-symmetric systems have different behaviors in case of environmental perturbations, including carrier wavelength drift, loop length change and temperature change. In this part, we investigate the effect of optical carrier wavelength drift on the operation of the PT-symmetric system.

First, we can assume that only one of the carrier wavelengths is perturbed, which is given by $\lambda_1 + \varepsilon_\lambda$. It can be derived from Eqs. (1) and (5) that the carrier wavelength perturbation will be transferred into the eigenvalue perturbation of the corresponding WSR, which is

$$\varepsilon_{\omega_m^{(1)}} = -\frac{2m\pi D}{\left[\tau_0 + D\cdot\left(\lambda_1 - \lambda_0\right)\right]^2}\varepsilon_\lambda \tag{21}$$

In our experiment, a 10-km standard ITU G.652 SMF is used, and the oscillating microwave signal has a frequency of 10 GHz. The approximate values for the quantities in (21) are $m = 4.8\times10^5$, $D = 170\ \text{ps/nm}$ and $\tau_0 + D\cdot\left(\lambda^{(1)} - \lambda_0\right) = 4.8\times10^{-4}\ \text{s}$. Substitute to Eq. (21), we have

$$\varepsilon_{\omega_m^{(1)}} = 2.2\times10^3\left(\text{rad/s}\times\text{nm}^{-1}\right)\cdot\varepsilon_\lambda \tag{22}$$

which indicates that for 1 nm of carrier wavelength drift, the eigenfrequency of the corresponding WSR will drift for 350 Hz (converted from angular frequency). The wavelength stability of the TLSs used in the experiment (Keysight N7714A) are 2.5 pm in 24 hours, indicating that the eigenfrequency drift will be less than 0.88 Hz, which is significantly less than the FSR of the WSRs or the oscillating frequency of the PT-symmetric system. Hence, optical carrier wavelength drift has small and negligible effect on the stable operation of the PT symmetric system. The WSR-based PT-symmetric OEO can operate with great stability.

For a PT-symmetric system in a critical state that $\gamma_m = \kappa$, the perturbed Hamiltonian can be written as

$$H = \begin{pmatrix} \omega_m + i\gamma_m + \varepsilon_{\omega_m^{(1)}} & -\kappa \\ -\kappa & \omega_m - i\gamma_m \end{pmatrix} \tag{23}$$

Letting $\det(H)=0$, we have

$$\omega_m = \frac{-\varepsilon_{\omega_m^{(1)}} \pm \sqrt{\varepsilon_{\omega_m^{(1)}}^2 + 4i\kappa\varepsilon_{\omega_m^{(1)}}}}{2} \tag{24}$$

Apparently, the wavelength perturbation on only one of the carrier wavelengths will increase the eigenmode mismatch between the two WSRs, induce an eigenvalue bifurcation of $\sqrt{\varepsilon_{\omega_m^{(1)}}^2 + 4i\kappa\varepsilon_{\omega_m^{(1)}}}$ and thus affects the stability of the PT symmetry operation of the system. Thus, it is important to use TLSs with good wavelength stability to ensure a small $\varepsilon_{\omega_m^{(1)}}$.

On the other hand, if both carrier wavelengths are perturbed for the same amount, the perturbed Hamiltonian will be

$$H = \begin{pmatrix} \omega_m + i\gamma_m + \varepsilon_{\omega_m^{(1)}} & -\kappa \\ -\kappa & \omega_m - i\gamma_m + \varepsilon_{\omega_m^{(1)}} \end{pmatrix} \tag{25}$$

Letting $\det(H)=0$ gives

$$\omega_m = -\varepsilon_{\omega_m^{(1)}} \tag{26}$$

The unique solution indicates that the no eigenvalue bifurcation will take place in this situation. The PT symmetry will be preserved with good stability as the eigenvalues of the two WSRs are shifting for the same amount toward the same direction, ensuring that the PT symmetry condition is always met.

Compare Eq. (23) with Eq. (25), the difference is that the perturbation is applied to one WSR for Eq. (23) and to both WSRs for Eq. (25). The combination of the two kinds of perturbation can represent any realistic perturbation that a PT-symmetric system may experience, as a perturbation can always be decomposed to common mode perturbation and differential mode perturbation.

Supplementary Note 6. STABILITY UNDER AMBIENT TEMPERATURE AND VIBRATION PERTURBATION

For low phase noise microwave signal generation, a PT-symmetric OEO is usually required to have a long loop length, which will result in a low phase noise. A dual-spatial loop PT-symmetric system can effectively solve the mode-selection problem for a long loop that has a small FSR (*2*). However, the dual-spatial loop architecture also makes the system more susceptible to ambient temperature or vibration interferences, which will cause eigenmode mismatch between the two subsystems. In this part, we investigate the response of the wavelength-space PT-symmetric oscillator to the environment interference and verify that a

wavelength-space PT-symmetric system has better stability compare to a spatial PT-symmetric system.

The ambient temperature and vibration interferences induce time-varying optical lengths for both WSRs, which arises due to the physical elongation of fiber and the photoelastic effect for vibration or strain interferences, and due to thermal expansion of fiber and the temperature-dependent refractive index for temperature interferences. The combined effects are the change of the optical length of the SMF. Without losing generality, we can assume that the length of the optical fiber remains constant and attributes all effects induced by temperature change and vibration to the change of effective refractive index of the SMF. Thus, the interference-induced refractive change can be written as (*5*):

$$\frac{\varepsilon_{n_{\text{eff}}}}{n_{\text{eff}}}=\left[1-\frac{n_{\text{eff}}^{2}}{2}\left(P_{12}-\nu P_{11}-\nu P_{12}\right)\right]\varepsilon_{\text{s}}+\left[\alpha+\frac{1}{n_{\text{eff}}}\frac{dn_{\text{eff}}}{dT}\right]\varepsilon_{\text{T}} \tag{27}$$

where ε_{s} and ε_{T} denotes the magnitudes of strain and temperature disturbance, respectively; $P_{i,j}$ are the Pockel's coefficients of the stress-optic tensor; ν is the Poison's ratio and α is the coefficient of the thermal expansion of silica. For our specific situation where the SMF is a standard G.652 silica fiber with an optical carrier wavelength of 1550 nm at a room temperature, the factors in Eq. (27) can be calculated, given by

$$\frac{\varepsilon_{n_{\text{eff}}}}{n_{\text{eff}}}=k_{\text{s}}\cdot\varepsilon_{\text{s}}+k_{\text{T}}\cdot\varepsilon_{\text{T}} \tag{28}$$

where we have $k_{\text{s}}=0.78\times10^{-6}\,\mu\varepsilon^{-1}$ and $k_{\text{T}}=6.67\times10^{-6}\,{}^{\circ}\text{C}^{-1}$ for silica fibers. The change of effective refractive index of SMF leads to a drift of eigenfrequency of the WSR. Substitute Eqs. (1) and (5) to Eq. (28), we have

$$\begin{aligned}\varepsilon_{\omega_m^{(1)}}&=-\frac{2m\pi c}{n_{\text{eff}}^{2}L}\varepsilon_{n_{\text{eff}}}\\&=-\frac{2m\pi c}{n_{\text{eff}}L}\left(k_{\text{s}}\cdot\varepsilon_{\text{s}}+k_{\text{T}}\cdot\varepsilon_{\text{T}}\right)\end{aligned} \tag{29}$$

where L is the length of the SMF and c is the light velocity in vacuum. In a laboratory environment, it is reasonable to assume disturbance magnitudes of $\varepsilon_{\text{s}}=1\,\mu\varepsilon$ and $k_{\text{T}}=0.1\,{}^{\circ}\text{C}$. The corresponding eigenfrequency shifts are 7.8 kHz and 6.7 kHz, respectively, which are in the same order of magnitude compared to the FSR of the WSRs and thus will have nonnegligible effect on the operation of the PT symmetry. Specifically, for PT symmetry based on spatial duplex architecture (*2, 3, 6, 7*), temperature and vibration affect two spatial loops differently, and thus are differential mode perturbations. The perturbed Hamiltonian and eigenfrequencies are given by Eqs. (23) and (24), respectively. With $\varepsilon_{\omega_m^{(1)}}$ in the order of several kHz, the

eigenfrequencies of supermodes experience strong bifurcation, which could lead to the breaking of PT symmetry. On the other hand, for the WSR-based PT-symmetric architecture, both PT-symmetric subspaces are enclosed in the same spatial loop. Temperature and vibration perturbations are applied to the two WSRs identically, and thus are common mode perturbations. Hence, the perturbed Hamiltonian and eigenfrequencies are given by Eqs. (25) and (26), respectively. No eigenfrequency bifurcation will be observed. Instead, the degenerate eigenfrequency shifts toward the same direction, indicating that the operation of PT symmetry is more stable in a WSR-based single spatial loop architecture.

Supplementary Note 7. EFFECT OF TEMPERATURE-DEPENDENT CHROMATIC DISPERSION

In the above Section, we proved that WSR-based PT-symmetric system is immune to ambient temperature disturbance that is introduced via thermal expansion or the temperature-dependent refractive index. In our experiment, the eigenmode alignments between the two WSRs are achieve by tuning the wavelength spacing between the two optical carriers according to the dispersion coefficient of the SMF. Note that the dispersion of an SMF is temperature-dependent; the temperature disturbance can also affect the PT symmetry operation by changing the dispersion. For the SMF with a fiber type of NZ-DSF, the thermal coefficient describing the relationship between chromatic dispersion and temperature is approximately given by (*8*)

$$\frac{1}{L}\cdot\frac{dD}{dT}=0.0025\ \text{ps/nm/km/°C} \tag{30}$$

In our experiment, the length of the SMF is 10 km and the magnitude of temperature disturbance is assumed to be 0.1 °C. We calculate the resulted chromatic dispersion perturbation to be $\varepsilon_{\text{D}}=0.0025\ \text{ps/nm}$. For carrier wavelength spacing of 2.353 nm that is chosen based on Eq. (6), the change of dispersion will lead to a mismatch between the two WSRs. Again, substitute Eq. (30) to Eqs. (1) and (5), we have

$$\frac{\varepsilon_{\omega_m^{(1)}}}{\varepsilon_{\text{D}}}=-\frac{2m\pi\Delta\lambda}{\left[\tau_0+D\cdot\Delta\lambda\right]^2}=3.08\times10^3\,\text{rad/s}\cdot\text{nm/ps} \tag{31}$$

and the eigenfrequency perturbation $\varepsilon_{\omega_m^{(1)}}$ is 7.7 rad/s or 1.2 Hz, which is small and negligible compared to the FSR of the WSRs or the oscillating frequency. The temperature-dependent chromatic dispersion affects the two WSRs differently as the carriers are of different wavelengths, and thus is a differential mode perturbation. The perturbed Hamiltonian and the eigenfrequencies should have the form of Eqs. (23) and (24), respectively. Although eigenfrequency bifurcation is present in this situation, the magnitude of eigenfrequency perturbation $\varepsilon_{\omega_m^{(1)}}$ is only in the order of 1 Hz, which is more than three order of magnitude lower than the 7.8 and 6.7 kHz eigenfrequency perturbation that temperature and vibration can introduce to a spatial PT-symmetric system, i.e., a PT-symmetric system in the wavelength space

can be over 1000 times more resilient to environmental perturbation compared to its spatial counterpart.

SUPPLEMENTARY REFERENCES

1. H. Hodaei *et al.*, Enhanced sensitivity at higher-order exceptional points. *Nature* **548**, 187 (2017).
2. J. Zhang, J. Yao, Parity-time–symmetric optoelectronic oscillator. *Sci. Adv.* **4**, eaar6782 (2018).
3. Y. Liu *et al.*, Observation of parity-time symmetry in microwave photonics. *Light Sci. Appl.* **7**, 38 (2018).
4. J. M. Senior, M. Y. Jamro, *Optical fiber communications: principles and practice*. (Pearson Education, 2009).
5. A. D. Kersey *et al.*, Fiber grating sensors. *J. lightwave Technol.* **15**, 1442-1463 (1997).
6. L. Feng, Z. J. Wong, R.-M. Ma, Y. Wang, X. Zhang, Single-mode laser by parity-time symmetry breaking. *Science* **346**, 972-975 (2014).
7. H. Hodaei, M.-A. Miri, M. Heinrich, D. N. Christodoulides, M. Khajavikhan, Parity-time–symmetric microring lasers. *Science* **346**, 975-978 (2014).
8. T. Kato, Y. Koyano, M. Nishimura, Temperature dependence of chromatic dispersion in various types of optical fiber. *Opt. Lett.* **25**, 1156-1158 (2000).

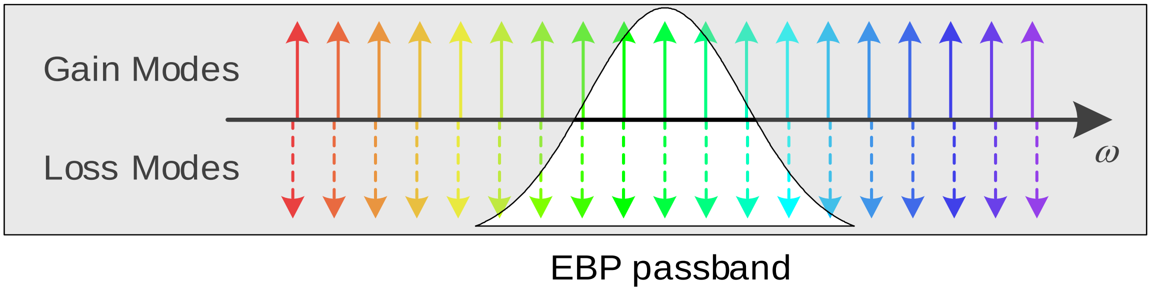

Supplementary Figure 1. Alignment of the eigenfrequencies of the gain and loss modes in the two WSRs. The tuning of carrier wavelength in conjunction with the chromatic dispersion in the SMF are adopted to align the eigenmodes between the two resonators.

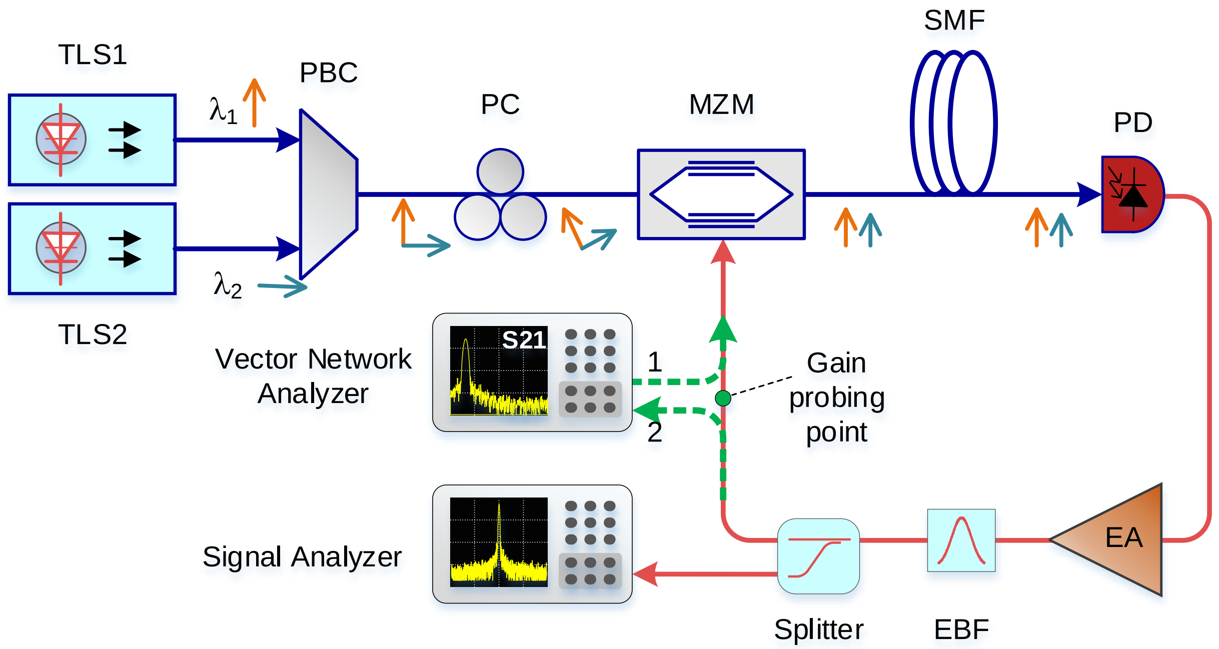

Supplementary Figure **2. Gain and loss coefficients probing and tuning in the WSRs.** A Vector Network Analyzer is deployed at the gain probing point between the microwave power splitter and the MZM. The measured loop gain coefficient, or S21 coefficient, is proportional to the square of the power of the optical carrier.

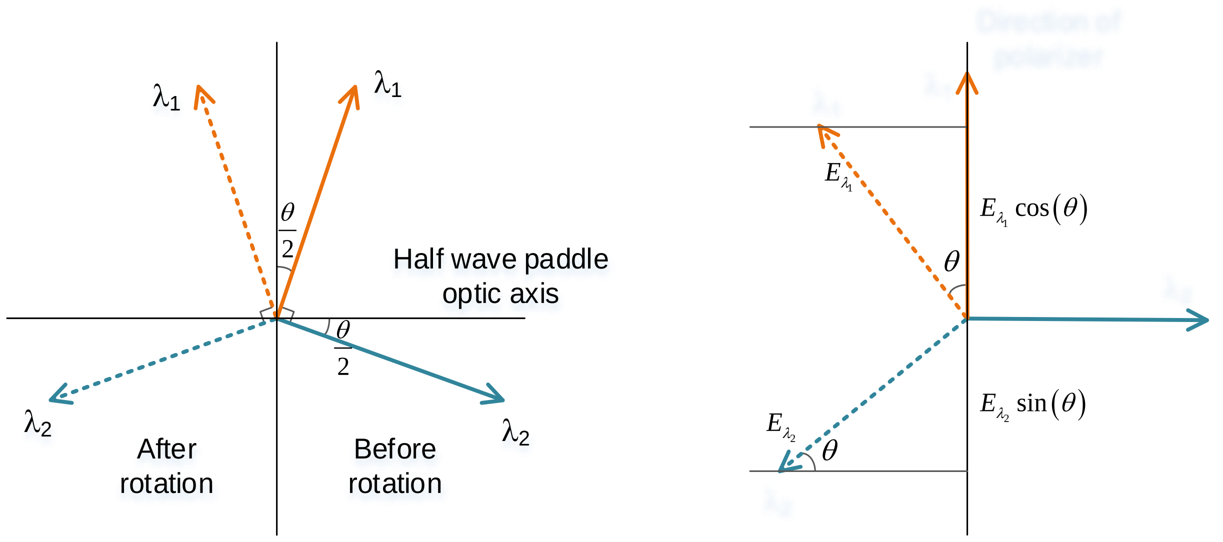

Supplementary Figure **3. Tuning PC changes carrier power and is used to fine-tune the gain and loss coefficients in the two WSRs.** a. Polarization rotation with the PC; b. carrier power tuning after propagating through a polarizer and polarization rotation using the PC.

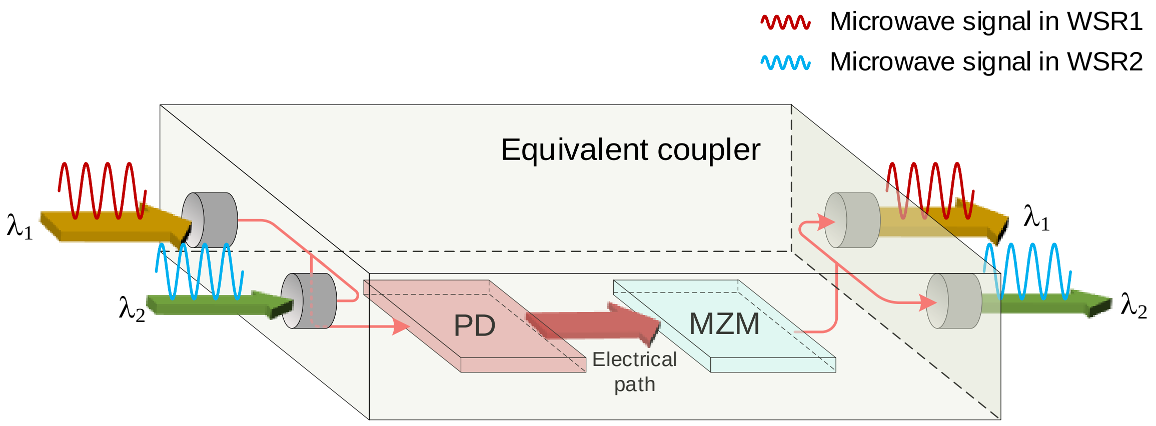

Supplementary Figure **4. The electrical path in the WSRs operating equivalently to a microwave coupler.** The microwave signal is combined and distributed indistinctively from and to the two carriers. The electrical path can be modelled as a coupler with a coupling ratio of 1:1, and the power of optical carrier is interpreted as the microwave insertion loss in the optical path in the WSRs.
